# Non-human immunodeficiency virus-related Kaposi’s sarcoma of the oropharynx: a case report and review of the literature

**DOI:** 10.1186/1752-1947-7-293

**Published:** 2013-12-31

**Authors:** Erika Crosetti, Giovanni Succo

**Affiliations:** 1 ENT Department, Martini Hospital, Turin, Italy; 2 ENT Department, S. Luigi Gonzaga Hospital, University of Turin, Turin, Italy

**Keywords:** HIV infection, Kaposi’s sarcoma, Oropharynx

## Abstract

**Introduction:**

Kaposi’s sarcoma is a malignant, slowly progressing, mesenchymal neoplasm characterized by a proliferation of connective tissue and capillaries. Clinical presentation is usually as nodules and red-purple plaques. This case report not only represents an uncommon presentation of Kaposi’s sarcoma in a non-immunocompromised patient, but also supports the role of viral infection in the pathogenesis of this disease. It provides some interesting information about this rare disease, particularly in patients who are human immunodeficiency virus negative.

**Case presentation:**

A 48-year-old Caucasian man presented with a sensation of a foreign body in his throat, accompanied by stomatolalia. Maxillofacial and neck magnetic resonance imaging confirmed the presence of a voluminous solid mass at the base of his tongue with oropharyngeal space reduction. Histological analysis indicated that the lesion was compatible with ulcerated Kaposi’s sarcoma of the oropharynx. Results of serological tests for human immunodeficiency virus infection were negative as was the result of the human herpesvirus-8 test, but the cytomegalovirus test result was positive.

**Conclusions:**

This case is unusual because the patient had only oropharyngeal localization of disease, without evidence of immunosuppression or the typical background or risk factors suggesting the classic or endemic form of Kaposi’s sarcoma. Isolated cases of Kaposi’s sarcoma with oropharyngeal manifestations not associated with human immunodeficiency virus infection are rare, and only 15 cases have been reported to date. At present, its localization, microscopic and histological characteristics, and patterns of progression are the main tools used for differential diagnosis of Kaposi’s sarcoma from other vascular neoplasms.

## Introduction

Kaposi’s sarcoma, the most common neoplasm associated with acquired immunodeficiency syndrome (AIDS), is a malignant, slowly progressing, mesenchymal neoplasm characterized by proliferation of connective tissue and capillaries. Clinical presentation is usually as nodules and red-purple plaques [1]. Here we present a case of Kaposi’s sarcoma of the oropharynx that was unrelated to human immunodeficiency virus (HIV) infection. Isolated cases of Kaposi’s sarcoma with oropharyngeal manifestations not associated with HIV infection are rare, and only 15 cases have been reported to date [2,3].

## Case presentation

**Figure 1:**
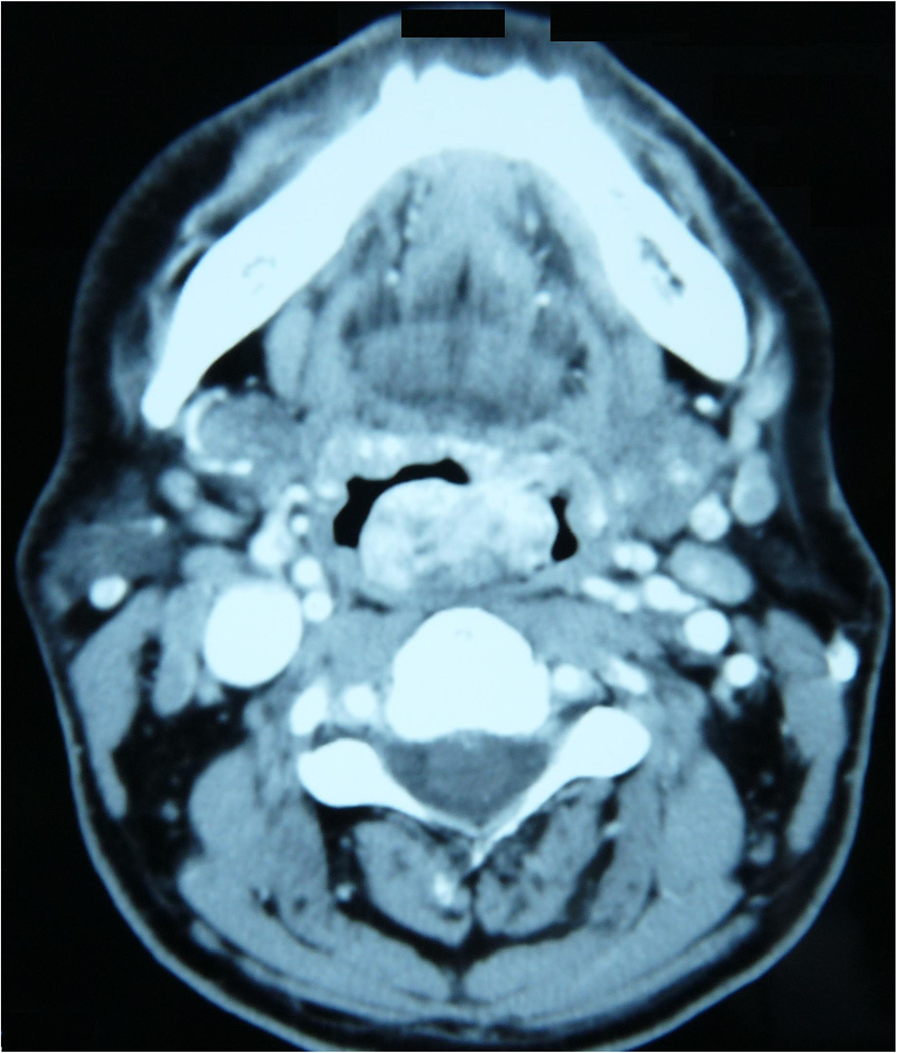
Maxillofacial and neck magnetic resonance imaging (axial view): presence of a voluminous solid mass at the base of the tongue with oropharyngeal space reduction.

**Figure 2:**
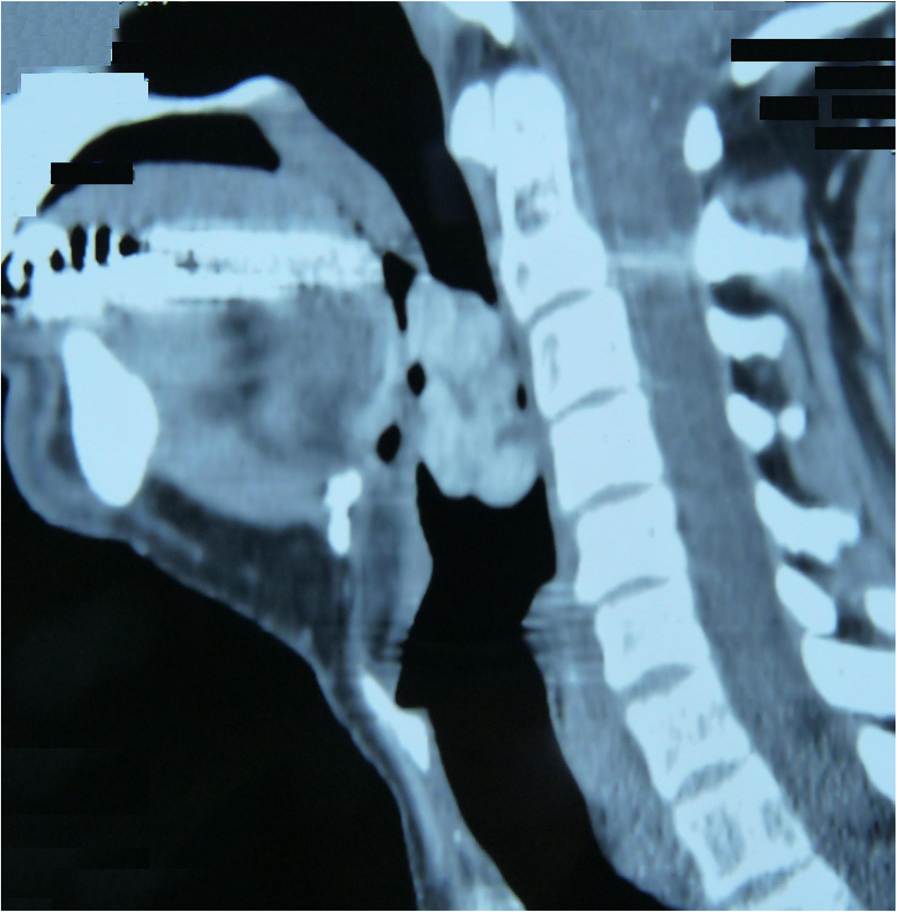
Maxillofacial and neck magnetic resonance imaging (sagittal view): voluminous mass at the base of the tongue.

A 48-year-old Caucasian man presented to our department referring the sensation of a foreign body in his throat, accompanied by stomatolalia. His family came from Sardinia. The man, a clerk, did not smoke and drank only socially. He was otherwise in good general health. An endoscopic examination showed the presence of a voluminous red-purple lesion at the base of his tongue, mobile, and reducing his oropharyngeal airway. Maxillofacial and neck magnetic resonance imaging confirmed the presence of a voluminous solid mass at the base of his tongue with oropharyngeal space reduction (Figures 1 and 2). He was subjected to direct microlaryngoscopy and carbon dioxide (CO_2_) laser excision of the mass. The surgical margins were negative. Histological analysis indicated that the lesion was compatible with ulcerated Kaposi’s sarcoma of the oropharynx. The postoperative period was uneventful. The patient was married, had regular sexual activity with his wife and reported that he did not practice oral sex. He also denied any intravenous drug abuse and he had never received immunosuppressive therapy. The results of serological tests for HIV were negative, and the patient also underwent dermatological examination. Clinical and radiological examination did not reveal any other localizations of disease. The human herpesvirus-8 (HHV8) test result was negative, but the cytomegalovirus test result was positive. He has undergone regular follow-up and is disease-free at the present time.

## Discussion

Kaposi’s sarcoma was described for the first time in 1872 by Moritz Kaposi using the term “multiple idiopathic hemorrhagic sarcoma” [1]. This disease is characterized by a variety of clinical and histological patterns. It is a malignant neoplasm that follows an indolent and usually protracted course. Kaposi’s sarcoma is generally classified into four forms [2-5]: classic or endemic; African; epidemic; or associated with renal transplantation.

The classic or endemic form usually affects males (male to female ratio is 15:1) who live in Mediterranean countries with a peak incidence between 50 and 70 years of age. Clinically, macular lesions are observed at the level of the trunk and inferior limbs, with a tendency to manifest as nodules and plaques. This form generally has a long clinical course (10 to 15 years) with slow progression, and patients often die of other causes. Its localization in the oral cavity and oropharynx is rare and occurs late in the course of disease [2,3]. In a review in 1975, Farman and Uys [6] identified 50 patients with an endoral localization of Kaposi’s sarcoma. These patients had the classic form of the disease, although diagnostic criteria were cutaneous localizations on the lower limbs.

The African form is typical in the central regions of Africa, and most frequently affects males between 25 and 40 years of age. Clinically, two subtypes have been identified [5]: a less aggressive type, characterized by the presence of plaques and cutaneous nodules with slow progression similar to the classic form and a more aggressive variety, which typically manifests in pediatric patients, in which there is visceral and lymph node involvement, in addition to classic cutaneous and mucosal lesions. This form has an extremely poor prognosis and patients usually die of gastrointestinal hemorrhage within 2 to 3 years after diagnosis. Oral and oropharyngeal localizations of the disease are rare in both subtypes of the African form [5].

The epidemic form is typical in patients with AIDS, and represents about 90% of malignant neoplasms diagnosed in these individuals. On clinical examination, the epidemic form is characterized by the appearance of disseminated mucocutaneous lesions associated with visceral and lymph node involvement. Localizations in the oral cavity and oropharynx are frequent and often represent the first symptom of disease. The clinical course of this form is dismal, as patients die quickly either from progression of disease or secondary complications associated with immunodeficiency [5-7].

In 1991, Chockley and Coke [8] reported that, in patients with AIDS and Kaposi’s sarcoma, the presence of localizations in the oral cavity and oropharynx were probably associated with progression of disease. In 77% of cases, localizations were observed on the palate, on the gums in 36%, and on the dorsal surface of the tongue in 15%. The gingival mucous and lips are less frequently involved. Such lesions, initially asymptomatic, can become ulcerous and painful.

The form of Kaposi’s sarcoma associated with renal transplantation has an incidence of 0.4% in the USA. The clinical course shows slow progression, but can be rapidly fatal in some cases due to massive visceral involvement. The extent of disease is directly proportional to the degree of immunodeficiency. Localizations in the oral cavity or oropharynx are rare in this subtype [4,9].

On histological examination, Kaposi’s sarcoma is characterized by a rich cellular component without atypia and with vascular lacunae. The stroma, in which blood vessels are present, is rich in extravasated erythrocytes and hemosiderin deposits. The four clinical forms of the disease present with nearly identical histological characteristics [9].

This case is unusual because our patient had only oropharyngeal localization of disease, without evidence of immunosuppression or the typical background or risk factors suggesting the classic or endemic form of Kaposi’s sarcoma. In other cases described in the literature, the lesions generally were localized on the posterior wall of the pharynx and were small. In our case, the imaging showed the presence of a voluminous solid mass at the base of the tongue with oropharyngeal space reduction [2,3].

The etiology of Kaposi’s sarcoma is still widely debated, although a viral origin is the most commonly accepted hypothesis at present. According to some authors [10,11], Kaposi’s sarcoma is not a true neoplasm, but rather a hyperplastic reaction caused by angiogenetic factors released from either CD4+ lymphocytes or viruses. Recent studies have shown that there is a close correlation between Kaposi’s sarcoma and HHV8. In particular, the presence of genomic HHV8 deoxyribonucleic acid (DNA) is a diagnostic tool for differentiating this neoplasm from other vascular tumors, such as hemangioendothelioma, kaposiform hemangioendothelioma, angiosarcoma, fibrosarcoma, and arteriovenous malformations [12,13].

In 1998, Hisaoka *et al.*[14] evaluated 93 cases of benign and malignant vascular lesions. All patients had a diagnosis of Kaposi’s sarcoma and all were positive for HHV8. More recently, the identification of HHV8 as a possible etiologic factor has suggested the potential efficacy of antiviral agents such as protease inhibitors in the treatment of Kaposi’s sarcoma. In 1998, Benfield *et al.*[15] described three cases of Kaposi’s sarcoma in complete remission after administration of protease inhibitors.

In our patient, the result of the HHV8 test was negative, but the result of the cytomegalovirus test was positive. In the literature, there is good evidence that cytomegalovirus could play a role in the pathogenesis of this disease. DNA from the virus has been localized to the tumor cells of Kaposi’s sarcoma by *in situ* hybridization methods, in non-HIV-related Kaposi’s sarcoma cases as well as those associated with HIV infection [16-20]. The role of cytomegalovirus in tumorigenesis is accomplished by the integration of a portion of its DNA into the host genome and, possibly, by amplification or mutation of oncogenetic sequences, in keeping with traditional models of virally induced tumor production [21].

Furthermore, the patient’s family came from Sardinia, even though he had grown up in the Piedmont region of Italy. The frequency of classic (non-HIV-related) Kaposi’s sarcoma is high in Sardinia [22]. Cerimele *et al.* have found that the human leukocyte antigen (HLA)-DR5 allele is greatly overrepresented in Sardinians with Kaposi’s sarcoma (significance level of P <0.001). By contrast, the HLA-DR3 allele associated with autoimmune diseases such as diabetes mellitus, Sjögren’s syndrome, and celiac sprue [22], is uncommon, compared with the control population. To add further support to these observations, other authors have shown that human leukocyte antigen (HLA)-DR5 is also common in patients who have HIV with this neoplasm [23]. The absence of the HLA-D3 allele in patients affected by non-HIV-related Kaposi’s sarcoma may reflect the relative lack of an immune response as a factor in the pathogenesis of this tumor.

Regarding therapeutic planning, as Kaposi’s sarcoma is a systemic multifactorial disease, management is based on symptoms. In general, the majority of patients with non-HIV-related Kaposi’s sarcoma and oral and/or oropharynx localization do not require specific treatment if symptoms such as odynophagia, dysphagia, and hemorrhage at other sites are not present. There are several treatment options for patients with the classic form and oral and/or oropharynx localization: CO_2_ or argon laser to minimize the possibility of bleeding; radiotherapy, Kaposi’s sarcoma is relatively radiosensitive, requiring a dose less than 2000 rad; intralesional or systemic chemotherapy with vinblastine (alone or in combination with other chemotherapeutic drugs); topical treatments are generally well tolerated, inexpensive, and repeatable, thus avoiding systemic immunosuppression.

This case report not only represents an uncommon presentation of Kaposi’s sarcoma in a non-immunocompromised patient, but also supports the role of viral infection in the pathogenesis of this disease. Non-HIV-related Kaposi’s sarcoma is probably caused by a similar mechanism to AIDS-related Kaposi’s sarcoma, which is related in part to constitutive susceptibility to viral oncogenesis (perhaps linked to the human leukocyte antigen (HLA)-DR5 locus). Cytomegalovirus probably plays a role in the development of the disease, in concert, in most cases, with concomitant immunodeficiency.

## Conclusions

Cases of Kaposi’s sarcoma with non-HIV-related oropharyngeal manifestations are rare, and to date only 15 cases have been reported in the literature. At present, its localization, microscopic and histological characteristics, and patterns of progression are the main tools used for differential diagnosis of Kaposi’s sarcoma from other vascular neoplasms.

## Consent

Written informed consent was obtained from the patient for publication of this manuscript and accompanying images. A copy of the written consent is available for review by the Editor-in-Chief of this journal.

## Competing interests

Both authors declare that they have no competing interests.

## Authors’ contributions

EC visited the patient, diagnosed the disease and analyzed and interpreted the patient data with regard to the oncological disease. GS performed the operation and regularly visited the patient. Both authors read and approved the final manuscript.
